# Purinoceptor: a novel target for hypertension

**DOI:** 10.1007/s11302-022-09852-8

**Published:** 2022-02-18

**Authors:** Xuan Li, Li-juan Zhu, Jing Lv, Xin Cao

**Affiliations:** 1 School of Acupuncture and Tuina, International Collaborative Centre On Big Science Plan for Purine Signalling, Chengdu University of Traditional Chinese Medicine, Chengdu, 610075 China; 2 Acupuncture and Chronobiology Key Laboratory of Sichuan Province, Chengdu, 610075 China

**Keywords:** Hypertension, Purinergic signalling, Purinoceptor, Neurotransmitter, Endothelium

## Abstract

Hypertension is the leading cause of morbidity and mortality globally among all cardiovascular diseases. Purinergic signalling plays a crucial role in hypertension through the sympathetic nerve system, neurons in the brain stem, carotid body, endothelium, immune system, renin-angiotensin system, sodium excretion, epithelial sodium channel activity (ENaC), and renal autoregulation. Under hypertension, adenosine triphosphate (ATP) is released as a cotransmitter from the sympathetic nerve. It mediates vascular tone mainly through P2X1R activation on smooth muscle cells and activation of P2X4R and P2YR on endothelial cells and also via interaction with other purinoceptors, showing dual effects. P2Y1R is linked to neurogenic hypertension. P2X7R and P2Y11R are potential targets for immune-related hypertension. P2X3R located on the carotid body is the most promising novel therapeutic target for hypertension. A_1_R, A_2A_R, A_2B_R, and P2X7R are all related to renal autoregulation, which contribute to both renal damage and hypertension. The main focus is on the evidence addressing the involvement of purinoceptors in hypertension and therapeutic interventions.

## Introduction

Hypertension is the leading cause of morbidity and mortality globally among all cardiovascular diseases [1]. In 2020, 1.28 billion adults reportedly had hypertension, which made the health condition an issue of global concern [2, 3]. The pathophysiology of hypertension is complex. It involves the multi-interaction of the sympathetic nervous system, the renin–angiotensin–aldosterone system, sodium homeostasis regulation, endothelium, and immune system. The drugs for controlling hypertension include β-receptor blocker, angiotensin-converting enzyme inhibitor, angiotensin II receptor blocker, calcium-channel-blocker, and diuretics. However, despite the availability of multiple antihypertensive drugs, approximately 1 in 5 adults (21%) with hypertension is out of control due to side effects, intolerance, and poor efficacy. The poor efficacy might be that not all the pathophysiological mechanisms are neutralized by the conventional antihypertensive therapies currently available. Given the large population with uncontrolled hypertension and the complexity of pathogenesis, developing new antihypertensive agents to provide more choices for those people is vital.

Accumulating evidence indicates that purinergic signalling has shown great therapeutic potential for hypertension. Purinergic signalling, that is, ATP acting as an extracellular signalling molecule, was proposed as a cotransmitter in sympathetic nerves in 1972 by Dr. Burnstock [1]. It involves the activation of cell surface P1 and P2 receptors by extracellular nucleosides and nucleotides [1]. P1 receptors are classified into 4 subtypes, namely, A_1_R, A_2A_R, A_2B_R, and A_3_R, which can be activated by adenosine. P2 receptors include 7 P2X ion channel receptor subtypes (P2X1-7) and 8 P2Y G-protein-coupled receptor subtypes (P2Y1, P2Y2, P2Y4, P2Y6, P2Y11, P2Y12, P2Y13, and P2Y14), which can be activated by purine nucleotides (ATP, ADP) [1]. A previous review in 2017 by Dr. Burnstock [4] showed alteration of purinergic signalling contributes to hypertension in 6 different ways, including sympathetic nerve activities, endothelial cells, neurons in the brain stem, carotid body, inflammation, and renin-angiotensin system. Sympathetic nerve activities [5–7] and the renin-angiotensin system [5, 8, 9] directly control the BP by acting on peripheral resistance, blood volume, and cardiac output. Neurons in the brain stem [10] and carotid body [11] indirectly regulate the BP by triggering a systemic response. Endothelial cells [5] and inflammation [12] affect the BP mainly by regulation of peripheral resistance through releasing vasoactive cytokines. Apart from the aforementioned mechanisms, emerging evidences showed that purinergic signalling mediated hypertension via affecting sodium excretion [13], epithelial sodium channel (ENaC) [14] activity, and renal autoregulation [15, 16], which were associated with blood volume. This review summarizes important aspects of purine nucleotides and their receptors in the regulation of BP.

## Purines in the regulation of blood pressure

Purines are released from the peripheral nerve fibres, endothelium in the local blood vessels, and the central nervous system (CNS), which play significant roles in the regulation of BP.

### Purines in the peripheral nervous system

ATP is released as a cotransmitter from sympathetic nerves in the local blood vessels which significantly contributes to the constriction of the blood vessels. Under high pressure, ATP is the chief functional sympathetic neurotransmitter [17]. It is released from the vesicles with noradrenaline [18–21] and neuropeptide Y in peripheral sympathetic nerves. ATP binds to P2 receptors (mainly P2X1R, P2X2R, P2X4R, P2Y1R, P2Y2R, and P2Y6R) on the smooth muscle cells, leading to the constriction of the local blood vessels [22–24]. Higher levels of ATP and sympathetic nerve density were also observed in diet-induced obesity rats, which were susceptible to hypertension. This finding hints that purinergic hyperactivity is closely associated with hypertension [25]. Besides peripheral sympathetic nerves, ATP is also released from perivascular sensory-motor nerves as a cotransmitter, which leads to vasoconstriction by targeting P2XR. But the main neurotransmitter from perivascular sensory-motor nerves is calcitonin gene-related peptide, which mediate vasorelaxation. Thus, the vascular tone is an interaction between multi-neurotransmitters.

### Purines in endothelium cells of blood vessels

ATP is released from endothelium cells besides sympathetic nerves and sensory-motor nerves in the local blood vessels. In lumen of blood vessels, shear stress- and hypoxia-induced ATP is released mainly from the endothelium and erythrocytes. ATP acted on P2X4R and P2YR to produce nitric oxide (NO) and endothelium-derived hyperpolarizing factor, resulting in vasorelaxation [26–28]. This effect was abolished by degradation of ATP by ectonucleotidases. In the rat isolated mesenteric arterial bed, ATP acted on P2YR evoking the prolonged phase of endothelium-independent vasorelaxation and activating Na + /K + -ATPase and KATP channels [29]. In endothelial and smooth muscle cell surface, ATP, ADP, and UTP were hydrolyzed by ectonucleoside triphosphate diphosphohydrolase, and then ecto-5′-nucleotidase hydrolyzed AMP into adenosine. Adenosine is bound to A_2A_R and A_2B_R, leading to vasorelaxation [24]. At 10^−5^ mol/kg^−1^, adenosine could generate NO-dependent hypotensive activity [30]. Augmented contractile responses to UDP and UTP were seen in femoral arteries of spontaneously hypertensive rats (SHR) than in those of Wistar-Kyoto [31]. The nucleotide uridine adenosine tetraphosphate (Up4A) is a dinucleotide comprising purine and pyrimidine moieties. It is proposed as a novel endothelium-derived vasoconstrictive factor. It binds mainly to P2X1R, and also P2Y2R and P2Y4R. Up4A induced hypertension by generating vasoconstriction and renal dysfunction [32]. The circulating level of Up4A was higher in juvenile hypertensives than in controls, thus indicating that Up4A was highly implicated with the onset of juvenile hypertension [33]. In the deoxycorticosterone acetate (DOCA)-salt rats, Up4A-induced contraction was enhanced in isolated renal arteries. This might be attributed to enhanced P2YR signalling and activation of the extracellular regulated protein kinases pathway [34]. However, different vascular beds responded differently to Up4A. Up4A-induced contraction was increased in renal but not in pulmonary arteries from DOCA-salt hypertensive rats [35]. Also, different concentrations of Up4A resulted in different vascular responses. At a low concentration, Up4A induced vasoconstriction on the mouse aorta, but at a high concentration, it led to hypotension and electrolyte retention in rats [36]. When Up4A was applied for controlling hypertension, region- and concentration-specific effects should be cooperated.

### Purines in central nervous system

In the CNS, ATP increased the central sympathetic drive, causing an increased systemic BP [37–39]. In the process, ATP activated the P2 receptors and thus increasing the firing activity of the hypothalamic sympathetic neurons [40]. In the commissural nucleus tractus solitarii (NTS), simultaneous blockade of ionotropic glutamate receptors and P2 receptors caused a remarkable decrease in the pressor and bradycardic responses [41]. In addition, ATP in the NTS mediated hindlimb vasodilation in response to alerting-defense [42]. These abnormal purinergic neurotransmissions and enhanced sympathetic activity are significant physiological processes which demonstrate that purinergic signalling in the CNS may be a potential target for hypertension.

Collectively, the effect of ATP on BP is a complex mechanism mainly involving P2XR and P2YR activation on peripheral sympathetic systems, CNS, and endothelial and smooth muscle cells, in addition, also involving the interaction among them. Similar dual effects on blood vessels are observed in ADP, adenosine, UTP, UDP, and Up4A. Region- and concentration-specific effects should be coordinated when referred to regulate BP. Purines are widely distributed and multifunctional, making it difficult to manipulate to reduce BP in clinical practice.

## Purinoceptors in the regulation of blood pressure

### P1 receptors in the regulation of blood pressure

All P1 purinoceptors, namely, A_1_R, A_2A_R, A_2B_R, and A_3_R, are found in smooth muscle and endothelial cells of arteries, as well as in the kidney [43–45], of which A_2A_R and A_2B_R are most commonly expressed in smooth muscle and endothelial cells [24]. Notably, the distribution of P1 receptors along the arteries varies [46].

P1 purinoceptors play an important role in cardiovascular responses [30] and renal sodium homeostasis. The activation of peripheral A_1_R decreased BP [47]. A_1_^−/−^ mice showed an elevated BP [48–50], plasma renin [48, 49], and sodium excretion [49]. A higher BP in A_1_^−/−^ mice attributed to a deficiency of A_1_R on the sympathetic innervation, thus causing more noradrenaline in the synaptic cleft to be released [51]. However, on a high-salt diet, the A_1_^−/−^ mice showed a lower BP than wild-type (WT) [49, 52, 53]. During chronic salt loading, A_1_R was downregulated, leading to the insensitivity of the renal arterioles or tubules to adenosine. This process facilitated renal sodium and water excretion and maintained the fluid volume and arterial pressure [54]. The different responses of A_1_^−/−^ mice to different diets could be explained by the lack of A_1_R on the renal afferent arteriole blunted tubuloglomerular feedback responses. Similarly, the hypertensive responses to NO inhibition [55] and angiotensin II (ANG II) mediated by tubuloglomerular feedback responses [55, 56] were blunted in A_1_^−/−^ mice when compared with WT.

A_2_R include A_2A_R and A_2B_R subtypes. A_2A_R mediated vasodilation via producing NO in the endothelium [30, 57, 58]. Intraperitoneal injections of A_2A_R agonists decreased the BP in Sprague–Dawley rats [47]. A_2A_^−/−^ Dahl salt-sensitive rats had a higher mean BP than WT [59]. Moreover, in never-treated essential hypertensive patients, lower affinity, higher density, and impaired function of A_2A_R were presented [60]. Besides, activation of A_2A_R led to hypotension through dilating the preglomerular microvessels mediated by epoxyeicosatrienoic acids [61, 62]. Activation of A_2B_R by adenosine also induced vasodilation, but the extent was much greater than A_2A_R in mesenteric arteries [50]. Of note, A_2B_R agonist showed either anti- or pro-hypertensive effects based on the various pathogenic mechanisms that induced BP. In Dahl salt-sensitive rats, the activation of A_2B_R caused diuresis and natriuresis. The dysfunctional A_2B_R impaired sodium excretion and resulted in elevated BP. In contrast, Dahl salt-sensitive rats with ANG II-induced hypertension activation of A_2B_R further released catecholamines. This triggered a proinflammatory state within the kidneys and/or the vasculature and thus contributing to high BP [63]. It was remarkable that A_1_^−/−^ and A_2A_^−/−^ displayed heterogeneity in gender. A_1_^−/−^ and A_2A_^−/−^ female rats rather than male rats revealed distinct lower BP than WT on a 4% salt diet [59].

A_3_R is abundantly expressed in afferent arterioles and participate in vasodilatation [43]. In the ANG II-supported circulation of the pithed rat, activation of A_3_R led to hypotension [64]. No BP elevation was observed in A_3_^−/−^ mice, after uninephrectomy and chronic HS intake, but high BP was detected in WT mice [65]. The mechanism might be related to inhibiting Na^+^/H^+^ exchanger-3 by A_3_R, contributing to sodium and fluid balance [66]. A_3_R was upregulated significantly in the renal cortex and medulla in salt-loaded rats, which may be an intrarenal adaptive mechanism to chronic salt loading [54].

Adenosine receptors have been proven to improve some hypertension complications. Activation of A_2A_R improved cardiac dysfunction and decreased cardiomyocyte hypertrophy, cardiac inflammation, and fibrosis, possibly by increasing fibroblast growth factor 21 [67]. It suggests that A_2A_R may have great therapeutic potential for hypertensive heart disease. A_2B_R could ameliorate hypertension-induced social memory impairment in SHRs [68]. In uninephrectomy and chronic HS intake-induced hypertension models, severe pathological changes of heart and kidney were observed in WT mice, but not in A_3_^−/−^ mice. In addition, A_3_R deficiency avoided oxidative stress in the renal [65].

Pulmonary arterial hypertension (PAH) is a life-threatening disease characterized by increased pulmonary arterial pressure and pulmonary vascular resistance. P1 receptors have been demonstrated to be a potential target in PAH. Nonselective P1 receptors antagonist aminophylline could attenuate the pulmonary vasodilation to adenosine in lamb model with hypoxia [69]. Among P1 receptors, A_2A_R is a promising target for PAH. In rats with monocrotaline-induced PAH, A_2A_R agonists, LASSBio-1386 [70] and LASSBio-1359 [71, 72], improved structural and functional alterations in heart and pulmonary artery, whereas A_2A_^−/−^ mice showed PAH, pulmonary vascular constriction, and pulmonary artery remodelling compared to WT littermates [73, 74]. A_2B_R was also involved in the onset of pulmonary hypertension. In contrast to A_2A_R, A_2B_R expression was upregulated in pulmonary artery smooth muscle cells from idiopathic PAH. Activation of A_2B_R could aggravate pulmonary vascular remodelling [75]. A_2B_R antagonist GS-6201 and genetic removal A_2B_R alleviated bleomycin-induced pulmonary hypertension and vascular remodelling [76]. Currently, information regarding the role of A_1_R and A_3_R in PAH is limited.

### P2 receptors in the regulation of blood pressure

P2X4R, P2Y1R, P2Y2R, and P2Y11R are the most expressed P2 receptors in endothelial cells [77] mediating pathways related to vasodilation via releasing NO, endothelium-dependent hyperpolarizing factor, and tissue-type plasminogen activator. In addition, P2 receptors, including P2X5R, P2Y1R, P2Y4R, P2Y6R, and P2Y14R, are responsible for human mesenchymal stem cells differentiation toward endothelial cells [78]. Moreover, P2Y2R is demonstrated to be associated with endothelial sprouting, vascular tube formation [79], and regulate shear stress-induced cytoskeletal alterations in human umbilical vein endothelial cells [80]. The dysfunction of above-mentioned purinoceptors may lead to abnormal vascular function, subsequently increasing the susceptibility to hypertension and hypertension-induced vascular damage. What’s more, P2XR-mediated neurogenic contractions are the predominant vasoconstrictor of small and medium arteries, namely, resistance artery [81].

P2 receptors play an important role in regulating of BP by acting on blood vessels and the CNS and participating in renal autoregulation. P2 receptors constricted afferent and efferent arterioles in ANG II-dependent hypertension [82] and triggered renal inflammation [83]. In inner medullary collecting duct cells, polycystin-2 and P2 receptors in response to flow might produce hypertension via Ca^2+^-dependent signalling pathways and thereby stimulating the synthesis of endothelin-1, an inhibitor of Na^+^ and water reabsorption [84]. ATP activated both P2XR and P2YR, modulating the activity of neurons in the rostral ventrolateral medulla (RVLM) [37]. Compared with the normotensive Wistar rats, SHRs showed a higher vascular tone of pial vessels on the RVLM region, indicating an augmented activity of sympathoexcitatory neurons, and a possible constant rise in the BP [85]. Brainstem P2 receptors mediated the hypothalamic defense area-NTS-RVLM pathway to regulate hindlimb vascular vasodilation. This process was achieved through attenuating sympathetic tone and increasing catecholamine release [42]. In addition, P2XR was expressed on the ventrolateral medulla projecting paraventricular nucleus neuron. These receptors might play a vital role in regulating sympathetic outflow [86]. Therefore, purinergic receptors may represent new avenues for treating hypertension resulting from over-activation of the sympathetic nervous in the CNS.

### P2X1 receptors mediate vasoconstriction and renal injury

Stimulation of P2X1R mediated vasoconstriction in vascular smooth muscle cells (VSMCs) from human gastro-omental arteries [87]. In addition, P2X1R is activated by neurally released ATP mediated Ca^2+^ entering the smooth muscle cells, thus inducing the sympathetic neurogenic contraction. Arteries from P2X1^−/−^ mice failed to contractions with the administration of P2XR agonist, whereas arteries from WT showed strong contractions [88].

Impaired P2X1R resulted in the dysfunction of autoregulation and microvascular reactivity. This impairment would lead to hypertension-induced renal injuries [89, 90]. Attenuated afferent arteriolar responses to P2X1R were observed in ANG II-infused rats on the HS diet [90]. The activation of normalized P2X1R averted lymphocyte infiltration, improved autoregulation [91], and protected renal autoregulation from inflammatory cascades induced by hypertension in DOCA-salt rats [92]. The plasma level of Up4A was elevated in hypertensive patients. Up4A activated P2X1R leading to hypertension, hence, the vascular P2X1R activity rather than plasma Up4A level might determine the role of Up4A in hypertension [93]. In summary, P2X1R shows great potential in regulating vascular tone in hypertension, mediating Ca^2+^ influx-caused vasoconstriction in VSMCs, and improving hypertension-induced renal injuries.

### P2X3 receptors in the carotid body regulate blood pressure

The carotid body (CB) [11] is a potential novel target for hypertension. As a peripheral chemoreceptor located at the bifurcation of carotid arteries, CB is hypersensitive to arterial oxygen, carbon dioxide, and blood pH levels. Thus, the reflex ventilation, cardiovascular system, and humoral response are regulated by CB. Researches during the past several decades on CB indicated that peripheral chemoreflex sensitivity of CB affected sympathetic activity and further influenced sympathetic-mediated diseases, such as hypertension. A significant increase of the CB reflex sensitivity was observed in SHRs [94] and hypertension patients [95]. Morphologically, CBs grew larger under the condition of hypertension in humans and rats [96, 97]. CB denervation effectively prevented the development and progression of hypertension in both hypertensive rats and patients [94, 95]. A clinical study found that hyperoxia-induced deactivation of CB chemoreceptors acutely lowered the BP in hypertensive patients [98]. Moreover, unilateral CB resection lowered the BP and sympathetic activity in 8 out of 15 patients with drug-resistant hypertension [99]. Interestingly, purinergic signalling is related to the functions of CB. It was demonstrated that P2X2R and P2X3R were expressed in petrosal neurons and were involved in ATP-mediated hypoxic chemo-transmission of CB in rats [100]. P2X3 mRNA expression was upregulated in the chemoreceptive petrosal sensory neurons of SHRs. Both tonic drive and hyperreflexia were normalized by local administration of a highly selective P2X3R antagonist. In conscious SHRs, blockade of P2X3R resulted in the reduction of arterial pressure and basal sympathetic activity, and the normalization of CB hyperreflexia. But, the P2X3R blocker did not affect normotensive Wistar [94]. More recently, canine models with P2X3R deficiency on CBs were created. This model showed a decreased BP and normalized the sympatho-vagal balance [101], thus suggesting the importance of P2X3R in CBs to control BP and sympathetic activity.

Taken together, antagonism of P2X3R in the CB lowers BP especially, and non-invasive targeted therapies will be more acceptable and needed by patients with neurogenic hypertension, refractory hypertension, or drug-resistant hypertension. Therefore, further researches should be the focus on those therapies.

### P2X4 receptors regulate vasodilatation and ENaC activity

P2X4R is highly expressed in endothelial cells and VSMCs of human resistance arteries [87]. Similarly, P2X4^–/–^ mice presented higher BP than WT mice [102, 103]. A clinical study found that the missense Y315C variant (rs28360472) in P2X4R was significantly related to increased pulse pressure, which might be attributed to a reduction in P2X4R-mediated vasodilation [104]. P2X4R has been proposed to be involved in lowering BP in two ways. Firstly, P2X4R in the endothelium was activated in response to shear stress, resulting in Ca^2+^ transients, NO formation, and thus inducing vasodilation [102, 103, 105–108]. Secondly, P2X4R was regarded as apical Na^+^ sensors to control Na^+^ balance and BP by modulating ENaC activity in the collecting duct [103, 109]. ENaC is relevant to the Na^+^ reabsorption at the distal nephron and is of great importance for BP regulation.

#### P2X7 receptors mediate renal injury and inflammation-related hypertension

A couple of genetic researches have proven that P2X7R is linked to night-time diastolic BP [110]. In addition, P2X7 non-synonymous rs3751143 polymorphism was linked to reduced susceptibility to essential hypertension and its estimated haplotypes in Chinese postmenopausal women [111]. However, another study revealed that, in untreated newly diagnosed essential hypertensive patients, two P2X7 gene SNPs 489C > T and 1513A > C were independent of altered endothelial function and arterial stiffness [112]. The mechanisms underlying the relation between P2X7 gene SNPs and hypertension are not fully understood, which deserves further investigation.

P2X7R may participate in the vicious cycle of salt-sensitive hypertension and renal injury in the Dahl salt-sensitive rats. In vivo, blockade of P2X7R could prevent and improve salt-sensitive hypertension and renal injury [113]. Likely, blockade and knockdown of P2X7R showed lower BP in ANG II-treated and DOCA-salt treatment rats [114]. In addition, a lower urinary albumin excretion, but a higher creatinine clearance was detected in P2X7^−/−^ mice, suggesting the additional protective renal function of P2X7R [114]. Also, selective inhibitor of P2X7R reduced BP in the normotensive Fischer (F344) rats, which exerted vasodilation in renal vascular and a lower pressure diuresis threshold. This potent hypotension effect by P2X7R might be associated with sevenfold increased expression of preglomerular vasculature P2X7R gene in F344 rat vs. in Lewis rat in the endothelium [115].

Compared with WT mice, P2X7R-deficient mice showed lower BP, less renal interstitial fibrosis and infiltration of immune cells, and lower levels of interleukin-1 beta. In ren-2 transgenic hypertension rat model, the expression of P2X7R was upregulated in podocytes and endothelial and mesangial cells after glomerular injury [116]. This upregulation indicated that P2X7R might be related to renal vasoconstriction and tubulointerstitial inflammation [117].

P2X7R is highly expressed in immune cells, including antigen-presenting cells (APCs), T cells, mast cells, macrophages, and monocytes. Activation of P2X7R led to release of interleukin-1 beta and interleukin-18, resulting in the inflammatory response [118, 119]. As we know, plasma ATP is higher in patients with hypertension than in normotensive controls. ATP acted on P2X7R of APCs and increased the expression of CD86. The action of ATP triggered the hyperactivation of T cells and contributed to the immune-mediated pathologic changes associated with hypertensive disease. Hydrolyzing ATP or blocking the P2X7R eliminated hypertension-induced T cells hyperactivation [120]. In addition, pharmacologic or genetic blockade of P2X7R suppressed the progression of hypertension. These findings support that P2X7R on APCs and T cells may be a potential target for immune-mediated hypertension.

### P2Y2 receptors mediate endothelium-dependent vasorelaxation and suppress ENaC activity

P2Y2R acted on the vasculature and renal Na^+^ reabsorption, manifesting the great therapeutic potential in hypertension. Activation of P2Y2R contributed to an acute NO-independent reduction in BP and an increase in renal Na^+^ excretion [121]. P2Y2R was the major subtype leading to vasorelaxation in human endothelial cells [122]. ATP released from the perivascular nerves activated P2Y2-like receptors in the endothelium thus eliciting an endothelium-derived hyperpolarizing factor in small arteries [123]. P2Y2R and G proteins Gq/G_11_ is a vital endothelial mechano-signalling pathway needed for basal endothelial NO formation, vascular tone. This pathway mediated fluid shear stress-induced several endothelial responses, such as [Ca^2+^]_*i*_ transients, and activation of the endothelial NO synthase [124]. However, another research reported P2Y2R activation resulted in a biphasic BP response mediated not by endothelial NO, but by endothelial-derived hyperpolarization, which required functional KCa3.1 (intermediate-conductance calcium-activated potassium channels) and connexin 37 [125].

The apical ATP/UTP-P2Y2-receptor system is a significant regulator to suppress the ENaC open probability in response to an increase in Na^+^ intake, thereby regulating NaCl homeostasis and BP [126–130]. Pharmacogenetic technology was used in the renal tubule, as compelling evidence to demonstrate that selective activation of the P2Y2R and G_q_ signalling was adequate to renal salt excretion in principal cells [131]. Hyperactivity of ENaC caused by an absence of P2Y2R [109, 126, 128] elicited an increase in BP [128]. P2Y2^−/−^ mice showed salt-resistant hypertension, enhancing renal Na^+^ retention and water reabsorption [127]. However, the inhibition of ENaC was not mediated by P2Y2R in the intact rat [129]. This discrepancy between species should be considered.

Interestingly, in the absence of P2Y2R, P2Y2/4R agonist resulted in an acute increase in BP. It was possibly attributed to vasoconstriction mediated by P2Y4R. There seems to be a mutual antagonism of P2Y2R and P2Y4R [121].

#### Other purinoceptors, including P2Y1, P2Y11, and P2Y12 receptors, might regulate BP

P2Y1R within the CNS and peripheral nerves were implicated in BP modulation. The inhibition of P2Y1R in C1 neurons produced a decrease in peripheral chemoreceptor-mediated activation of phrenic nerve activity, sympathetic nerve activity, and BP [132]. In muscle afferents, inhibition of P2Y1R prevented increased mean BP induced by forced exercise in the ischemic injury mice. This antihypertensive effect might involve regulating membrane expression of acid-sensing ion channel 3 [133].

P2Y1R and P2Y2R were probably associated with salt-sensitive hypertension. On the HS diet, P2Y1R and P2Y2R in the inner medullary collecting duct cells participated in the adaptive mechanism for increasing urinary NaCl excretion [134].

Among the P2YR subtypes, P2Y6R is the highest expressed type in mouse resistance arteries [135]. Compared with WT, P2Y6^−/−^ mice had a lower BP. In vitro studies, P2Y6R activated by UDP and UTP were responsible for arterial contraction in the rat model. P2Y6R also participated in myogenic tone via an autocrine/paracrine activation loop [135]. P2Y11R is closely linked with inflammation and may have therapeutic potential in immune system-related hypertension. P2Y11R activation improved the vasomotor function and decreased hydrogen peroxide release, indicating a heightened role for P2Y11R in inflammation-related endothelial dysfunction [136]. P2Y12R may regulate BP for participation in the balance of body fluid. It partly mediated ADP-induced antihypertensive effect via decreasing tubular water reabsorption and urine concentration [137].

In addition, P2X1R, P2X7R, and AT1R seemed to share the same receptor or post-receptor signalling pathways [138]. In ANG II-dependent hypertension, P2XR and AT1R receptors mediated renal vasoconstriction, but P2XR was predominant [138, 139].

Altered P2 signalling also contributed to the development of PAH. In PAH, the protein of P2X1R, P2X4R, P2Y2R, P2Y11R, and P2Y12R were upregulated. P2X1R and P2Y12R mediated ATP-induced vasoconstriction, and P2Y6R mediated UDP-induced vasoconstriction in rat intrapulmonary arteries [140]. P2YR activated by Up4A resulted in pulmonary arteries contraction in rats [141, 142]. The P2 receptor-mediated Ca^2+^ signalosome of the human pulmonary endothelium, which was implications for PAH [143]. Shear stress modulated endothelial Kruppel-like factor 2 (KLF2) through activation of P2X4R [144], and KLF2 mutation presented heritable PAH in clinical cases [145]. In addition, modulation of KLF2 showed therapeutic potential for pulmonary hypertension [146]. Thus, targeting P2X4R may be an option for modulating KLF2 in the PAH.

In the animal models with PAH, inhibition of P2X7R attenuated the inflammation [147, 148], pulmonary arteries remodelling [147, 149], and right ventricular function [147, 148]. The blockade effect of P2X7R on PAH and RV complications differed from current treatment options, where the significant improvements in pulmonary pressures ultimately do not prevent mortality due to RV failure [148]. Blockade of P2Y1R also decreased pulmonary arterial pressure in swine with acute hypoxia-induced pulmonary hypertension [150].

Overall, P2X4R and P2X7R may be therapeutic options in PAH. More studies are needed further to elucidate the involvement of P2X4R and P2X7R in PAH.

## Conclusion and perspectives

Endogenous purines function as extracellular signalling molecules by activating purinoceptors. Purines and purinoceptors play a significant role in the regulation of BP. ATP mainly mediates vascular tone through the activation of P2X1R and P2X6R on the smooth muscle cells. ATP can also mediate vascular tone by activating A_2A_R, P2X4R, and P2Y2R on endothelial cells, and also by interacting with other purinoceptors, thereby showing dual effects. Similar dual effects are observed in ADP, adenosine, UTP, UDP, and Up4A.

**Fig. 1:**
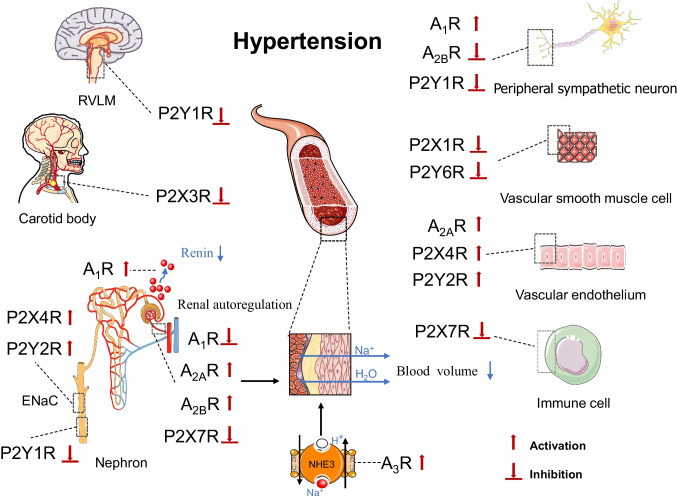
Purinoceptors in regulation of blood pressure. Activation or inhibition of purinoceptors can improve hypertension in 9 different ways, including sympathetic nerve activities, endothelial cells, neurons in the brain stem, carotid body, inflammation, renin-angiotensin system, sodium excretion, ENaC activity, and renal autoregulation. P2Y1R is involved in neurogenic hypertension. P2X3R located on the carotid body is the novel therapeutic target for hypertension. A_1_R activation induces renin release. A_1_R, A_2B_R_,_ A_3_R, P2X4R, P2Y1R, and P2Y2R mediate sodium excretion. P2X1R and P2X6R on the smooth muscle cells, and A_2A_R, P2X4R, and P2Y2R on endothelial cells act on vessels to regulate vascular tone

Purinoceptors play a crucial role in hypertension by modulating renin release, sodium excretion, ENaC activity and renal autoregulation, sympathetic nerve system, endothelium, CB, and immune system (Fig. 1). P2Y1R is demonstrated to be involved in neurogenic hypertension. P2X7R and P2Y11R are shown to be potential targets for immune-related hypertension. P2X3R located on CB is the most promising novel therapeutic target for hypertension. A_1_R, A_2A_R, A_2B_R, and P2X7R play significant roles in mediating renal autoregulation. The dysfunction of renal autoregulation eventually leads to renal damage and high BP. Purinergic signalling performs other roles which are similar to those of classic antihypertensive agents, including A_1_R, A_2B_R, A_3_R, P2X4R, P2Y1R, and P2Y2R mediated sodium excretion. A_1_R and P2X4R are related to NO formation. A_1_R activation induces renin release. Though numerous studies have demonstrated the effect of purines and purinoceptors on BP, none of the pharmacological tools targeting purinoceptors has entered clinical trials due to lower efficacy, complex kinetics issues, and adverse effect. Future studies are required to pinpoint purinoceptors and evaluate the purines and their receptors for potential treatment. More studies are needed to evaluate the purines and purinoceptors for the treatment of hypertension.

Collectively, there is a complexity of purines- and purinoceptor-mediated effects in BP. Many aspects are still not fully understood due to many discrepant observations. The discrepancy arises from (1) purines concentration vs. purinoceptors sensitivity and (2) differences in purinoceptor expression and distribution in different blood vessels of different species. A better understanding of these aspects will help elucidate the role of purines and purinoceptors in the regulation of BP and the development of novel therapeutic strategies.

Many questions remain from purinoceptors in the regulation of BP. Firstly, purines and purinoceptors are extensively distributed in the body. The BP regulation is a complex process mediated by purinoceptors in local blood vessels and the CNS. Thus, the assessment of drug safety and side effects should be of great concern. Secondly, heterogeneities in species, genders, and vascular beds should be taken into account. Lastly, the purinoceptors not mentioned above, including P2X2R, P2X5R, P2X6R, P2Y1R, P2Y3R, and P2Y13R, might have an impact on the regulation of BP.

## Data Availability

Not applicable.
