# Correction to: Purinoceptor: a novel target for hypertension

**DOI:** 10.1007/s11302-023-09927-0

**Published:** 2023-02-18

**Authors:** Xuan Li, Li-juan Zhu, Jing Lv, Xin Cao

**Affiliations:** 1 School of Acupuncture and Tuina, International Collaborative Centre On Big Science Plan for Purine Signalling, Chengdu University of Traditional Chinese Medicine, Chengdu, 610075 China; 2 Acupuncture and Chronobiology Key Laboratory of Sichuan Province, Chengdu, 610075 China

**Correction to: Purinergic Signalling**

10.1007/s11302-022-09852-8

The original version of the article unfortunately contained an error.

The funding number was inadvertently added and miswritten in the Funding. The correct one is shown below.

This work was supported by grant from the National Natural Science Foundation of China (81904306) and Sichuan Science and Technology Program (2020YFH0115).

The original article has been corrected.

